# Biocontrol of Sugarcane Smut Disease by Interference of Fungal Sexual Mating and Hyphal Growth Using a Bacterial Isolate

**DOI:** 10.3389/fmicb.2017.00778

**Published:** 2017-05-09

**Authors:** Shiyin Liu, Nuoqiao Lin, Yumei Chen, Zhibin Liang, Lisheng Liao, Mingfa Lv, Yufan Chen, Yingxin Tang, Fei He, Shaohua Chen, Jianuan Zhou, Lianhui Zhang

**Affiliations:** ^1^Integrative Microbiology Research Centre, South China Agricultural UniversityGuangzhou, China; ^2^Guangdong Province Key Laboratory of Microbial Signals and Disease Control, South China Agricultural UniversityGuangzhou, China; ^3^State Key Laboratory for Conservation and Utilization of Subtropical Agro-BioresourcesGuangzhou, China

**Keywords:** sugarcane smut, *Sporisorium scitamineum*, sporida, sexual mating, biocontrol

## Abstract

Sugarcane smut is a fungal disease caused by *Sporisorium scitamineum*, which can cause severe economic losses in sugarcane industry. The infection depends on the mating of bipolar sporida to form a dikaryon and develops into hyphae to penetrate the meristematic tissue of sugarcane. In this study, we set to isolate bacterial strains capable of blocking the fungal mating and evaluate their potential in control of sugarcane smut disease. A bacterial isolate ST4 from rhizosphere displayed potent inhibitory activity against the mating of *S. scitamineum* bipolar sporida and was selected for further study. Phylogenetic analyses and biochemical characterization showed that the isolate was most similar to *Pseudomonas guariconensis*. Methanol extracts from minimum and potato dextrose agar (PDA) agar medium, on which strain ST4 has grown, showed strong inhibitory activity on the sexual mating of *S. scitamineum* sporida, without killing the haploid cells MAT-1 or MAT-2. Further analysis showed that only glucose, but not sucrose, maltose, and fructose, could support strain ST4 to produce antagonistic chemicals. Consistent with the above findings, greenhouse trials showed that addition of 2% glucose to the bacterial inoculum significantly increased the strain ST4 biocontrol efficiency against sugarcane smut disease by 77% than the inoculum without glucose. The results from this study depict a new strategy to screen for biocontrol agents for control and prevention of the sugarcane smut disease.

## Introduction

Sugarcane smut disease caused by the fungal pathogen *Sporisorium scitamineum* is a devastating disease in sugarcane growing areas globally, which results in considerable loss of sugar yield (Comstock, 2000). In China, the average smut infection rate is over 10%, and can reach over 50% in some fields, causing billions economical loss every year (Shen et al., 2013). Currently, control of sugarcane smut disease mainly relies on the breeding of resistant cultivars (Shen, 2002; Wada, 2003; Croft et al., 2008; Lwin et al., 2012; Shen et al., 2014), which is constrained by long breeding processes, high cost, and availability of smut-resistant parental lines. To some extent, the disease could also be controlled by soaking seed canes using chemical fungicides (Olufolaji, 1993; Wada, 2003; Bhuiyan et al., 2012), or using plant or fungal extracts that inhibit the smut pathogen germination and growth (Lal et al., 2009). However, large scale application of toxic chemicals may cause environmental hazards and residual problems.

The pathogen produces two types of haploid sporida, which mate together to form a dikaryon, and then develops into hyphae to penetrate the bud scales of the sugarcane plant and infect host meristematic tissues (Croft and Braithwaite, 2006; Yan et al., 2016b). Therefore, sexual mating of the two sporida is of critical importance for fungal life cycle and the disease occurrence. In this study, we set to screen for bacterial isolates capable of inhibiting the mating of *S. scitamineum*. Among a few hundreds of the bacterial isolates tested, a strain identified as *Pseudomonas* sp. ST4 from vegetable rhizosphere in Shantou city, Guangdong province, China, was found secreting (a) metabolite(s) with potent activity in inhibiting the sexual mating of *S. scitamineum* without killing fungal haploid cells. Greenhouse trials showed that *Pseudomonas* sp. ST4 could be a promising biocontrol agent for effective control of sugarcane smut disease.

## Materials and Methods

### Microorganisms and Culture Conditions

Bacterial strains were grown in Luria-Bertani (LB; per liter contains 5 g yeast extract, 10 g peptone, and 10 g NaCl) or minimal medium broth (MM, per liter contains 10.5 g K_2_HPO_4_, 4.5 g KH_2_PO_4_, 2 g (NH_4_)_2_SO_4_, 2 g mannitol, 2 g glycerol, 0.2 g MgSO_4_ 7H_2_O, 5 mg FeSO_4_, 10 mg CaCl_2_, and 2 mg MnCl_2_, pH 7.0) or on solid medium containing 1.8% (wt/vol) agar at 28°C (Zhou et al., 2011; Liao et al., 2014). *S. scitamineum* haploid cell lines MAT-1 and MAT-2 were grown and maintained in YEPS medium (per liter contains 10 g yeast extract, 20 g peptone, and 20 g sucrose) at 28°C or on solid medium containing 1.8% (wt/vol) agar (Yan et al., 2016b). Potato dextrose agar (PDA, per liter contains 200 g potato, 20 g glucose, and 18 g agar) was used in bioassay of bacterial antagonistic activity against fungal mating. Potato agar (PA) medium is the same as PDA except lacking glucose.

### Bacterial Isolation and Screening for Inhibition Activity against the Sexual Mating of *S. scitamineum*

Over 3000 bacterial isolates were obtained from soil samples collected from five provinces of China, including Guangdong, Henan, Xinjiang, Jiangxi, and Guangxi, following a method described previously with minor modifications (Dong et al., 2000). Briefly, soil samples were suspended in sterilized water with shaking for 1 h before spreading on LB plates. Visibly distinct colonies from each sample were streaked onto plates to obtain single colonies to ensure the purity of the isolates. The single colonies were inoculated and cultured in LB liquid medium at 28°C with shaking overnight. For bioassay of inhibitory activity against the mating of *S. scitamineum*, the PDA plate was cut into separated slices (0.6 cm in width). An aliquot of 1 μl bacterial culture was added on one end of the agar slice, and then the mixture of *S. scitamineum* haploid cells MAT-1 and MAT-2 strain was spotted (0.5 μl of OD_600_ ≈ 1.5) on the slice at progressively further distances from the loaded sample. LB medium was added in the same way as bacterial culture as a negative control. The plates were incubated at 28°C for 2 days, until the white hypha in the negative control grew to reach the edges of the slice. The candidate isolates inhibiting hyphal growth but allowing haploid growth were selected for further analysis.

### Bioassay of Extracellular Metabolites from Strain ST4

As the inhibitory activity was hardly detectable in liquid culture supernatants, we produced ST4 extracellular metabolites using PDA agar plates. Briefly, strain ST4 cells (OD_600_ ≈ 1.5) were evenly spotted at about 1 cm distance on PDA plates. The plates were incubated at 28°C for 48 h, and bacterial cells were removed by peeling off the agar surface containing the bacterial colonies. The remaining PDA medium was melted at around 70°C, and mixed with fresh PDA medium at 1:1 ratio before pouring into a new petri dish. After solidification, a mixture of *S. scitamineum* haploid cells MAT-1 and MAT-2 was spotted (0.5 μl of OD_600_ ≈ 1.5) on the plate. A mixture spotted on a fresh PDA plate was used as a negative control. The plates were incubated at 28°C for 1–2 days, until white hypha appeared in the control plate.

### Impacts of ST4 Extracellular Metabolites on Mating and Hyphal Growth of *S. scitamineum*

To further determine whether ST4 extracellular metabolites affect sexual mating of compatible haploid cells, we tested cell fusion using MAT-1 and MAT-2, respectively, marked with GFP and RFP for visualization (Yan et al., 2016a). MAT-1, MAT-2 and their mixture were, respectively, spotted evenly in 1 cm-distance on plates of PDA or PDA+ST4 metabolites, and grown for 2–3 days. Morphology of bipolar sporida and hyphae was observed under 100× magnification using ZEISS Observer. Z1 and mating rate was measured. To test the impacts of ST4 metabolites on hyphal growth of the resulting dikaryon, firstly, we spotted the mixture of MAT-1 and MAT-2 on PDA plates and incubated for 12, 16, 20, 24, 28, and 48 h. Secondly, hyphae were transferred to new plates of PDA or PDA+ST4 metabolites, and grown for 2–3 days to observe hyphal morphology.

### Medium and Sugar Effect on the Inhibitory Activity of Strain ST4

For identification of suitable medium to support production of fungal mating-inhibitory compounds, strain ST4 was spotted as described in previous section on the agar slice of the following media including LB, MM, PDA, and PA, with or without glucose 2% (wt/vol) as indicated. For testing the effect of different sugars and derivatives on the inhibitory activity of strain ST4 against the sexual mating of *S. scitamineum*, the PA medium supplemented with various sugars or derivatives as indicated at a final concentration of 2% or otherwise as indicated.

### Greenhouse Trial

*Sporisorium scitamineum* haploid strains MAT-1 and MAT-2 were, respectively, inoculated into 100 ml YEPS liquid medium at 28°C, shaking at 200 rpm for 2 days. Strain ST4 was cultured in 100 ml LB medium at 28°C, shaking at 200 rpm for 12 h. Cells were collected by centrifuge at 3000 rpm for 10 min. Collected cells were washed twice with PBS buffer and re-suspended in PBS buffer (OD_600_ ≈ 3.0). The above three fungal and bacterial strains were mixed at 1:1:1 ratio with or without glucose (2%, wt/vol), and an aliquot of 1 mL of treatment was inoculated by injection to the seedling (around four leaves) stem base of sugarcane cultivar ROC22, which is susceptible to *S. scitamineum*. Sugarcane seedlings were inoculated with the mixture of two fungal haploid strains as a positive control, and same amount of PBS buffer as a negative control. Thirty seedlings were used in each treatment and the trial was performed twice.

### DNA Extraction and PCR Amplification

Bacterial genomic DNA was extracted using the MasterPure^TM^ DNA Purification Kit following the manufacturer’s protocol (Epicentre Co., USA). Primers used for amplification of the 16S rRNA gene were 27F and 1492R with conditions determined by Coenye et al. (1999). Amplification and partial sequencing of the DNA gyrase subunit B (*gyrB*), RNA polymerase β subunit (*rpoB*), and RNA polymerase subunit D (*rpoD*) genes were performed as described (Mulet et al., 2010), using the primers GyrBPUN1F/GyrBPUN1R for *gyrB* (Ramos et al., 2013; Toro et al., 2013), LAPS5F/LAPS27R for *rpoB* (Ait et al., 2005) and PsEG30F/PsEG790R for *rpoD* (Mulet et al., 2009). Amplicons were purified with Omega’s E.Z.N.A. TM Cycle Pure Kit following the manufacturer’s protocol (Omega, USA), and sequenced by Invitrogen Company in Guangzhou, China. The sizes of the resultant amplicons were: *gyrB* = 752 bp, *rpoB* = 1230 bp, and *rpoD* = 746 bp.

### Multilocus Sequences Analysis (MLSA) and Phylogenetic Analysis

Sequences analyses were performed using DNASTAR Lasergene SEQMAN program. Sequence similarities of 16S rRNA gene were determined using EzTaxon-eserver^1^ (Kim et al., 2012) and BLASTn program. Sequences of *gyrB*, *rpoB*, and *rpoD* genes of related strains were obtained from GenBank database. Sequences were aligned with ClustalW and phylogenetic trees were reconstructed using the neighbor-joining method with maximum composite likelihood model. Bootstrap analysis was performed based on 1000 replicates. The MEGA5 package was used for all the phylogenetic analyses (Tamura et al., 2011). The GenBank accession numbers of the 16S rRNA, *gyrB*, *rpoB*, and *rpoD* gene sequences of strain ST4 are KU496562, KU496563, KU496564, and KU496565, respectively.

### Colonial and Microscopic Morphology

Colonial morphology was observed on LB agar plate 8 h after inoculation and incubation at 28°C. The bacterial cells were suspended in sterile distilled water and stained with phosphotungstic acid [3% (v/V), pH 7.0] for 2 min, air-dried and observed by using transmission electron microscope (Hitachi H7650).

### Biochemical Test

Phenotypic characterization was carried out according to the minimal standards proposed by Toro et al. (2013). Biochemical features of the isolate were studied using standardized procedures (Grimont and Grimont, 2005), including the following tests: Gram staining, oxidase, catalase, motility, utilization of oxygen, glycerol fermentation test, gas and acid production from glucose, nitrate reduction, salt tolerance and hemolysis. For fluorescent pigment analysis, cells were grown in King’s B agar at 30°C, and testing for pigment production using a visible-UV on 1, 3, and 5 days. Additionally API 20NE and Biolog GEN III Microplates (Biolog) were used following the manufacturers’ instructions. The results from API 20NE was recorded after 48 h incubation at 28°C. The Biolog system (GEN III v2.7.1.40.I5G) was used to determine the carbohydrate fermentation profile and readings were made after 22 h of incubation at 33°C.

## Results

### Strain ST4 Produces Inhibitory Metabolites against the Sexual Mating of *S. scitamineum*

**FIGURE 1:**
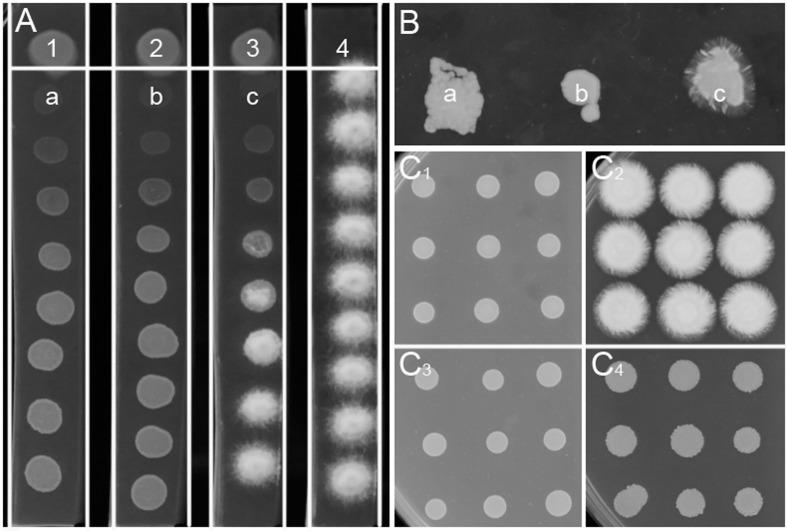
**Inhibitory effect of strain ST4 and its metabolites on sexual mating of *S. scitamineum.*** **(A)** Dual culture of ST4 and *S. scitamineum* haploid cells MAT-1 or MAT-2. 1, ST4 and MAT-1; 2, ST4 and MAT-2; 3, ST4, MAT-1 and MAT-2 mixture; 4, MAT-1 and MAT-2 mixture; **(B)** a, b, c grow on the new PDA plate; **(C_1_)** Mixture MAT-1 and MAT-2 on the metabolite plate of ST4; **(C_2_)** Mixture MAT-1 and MAT-2 on the PDA plate; **(C_3_)** MAT-1 or MAT-2 on the metabolite plate of ST4; **(C_4_)** MAT-1 or MAT-2 on the PDA plate.

More than 3000 bacterial isolates were isolated from the rhizospheres of sugarcane, vegetables, bamboo, maize, and many other plants from five provinces in China, including Guangdong, Henan, Xinjiang, Jiangxi, and Guangxi. These bacterial isolates were spotted onto PDA plates for screening of bacterial antagonists against the sexual mating of *S. scitamineum*. A total of 68 isolates showed different levels of inhibitory effect on the hyphal growth of *S. scitamineum*, but for most of them, this effect was due to killing or stopping the growth of haploid cells rather than direct interference of the fungal sexual mating, which was confirmed by using ST4 metabolite plate assay (data not shown). Bacterial isolate ST4, which showed a strong activity to inhibit the hyphal growth of mixed *S. scitamineum* cells (Supplementary Figure S1), was selected for further analysis in this study. The results showed that the fungal cells at the closest vicinity of strain ST4 (MAT-1, MAT-2, and MAT-1/MAT-2 mixture, labeled as a, b, c, respectively, at **Figure 1A**) grew poorer than the cells at further distance. These fungal cells at a, b, and c were re-inoculated onto a fresh PDA plate, and the results showed that the haploid cells grew up normally (a and b in **Figure 1B**) and the mixture cells produced hyphal cells (c in **Figure 1B**), indicating that the diffusible metabolites from strain ST4 didn’t kill the fungal cells.

**FIGURE 2:**
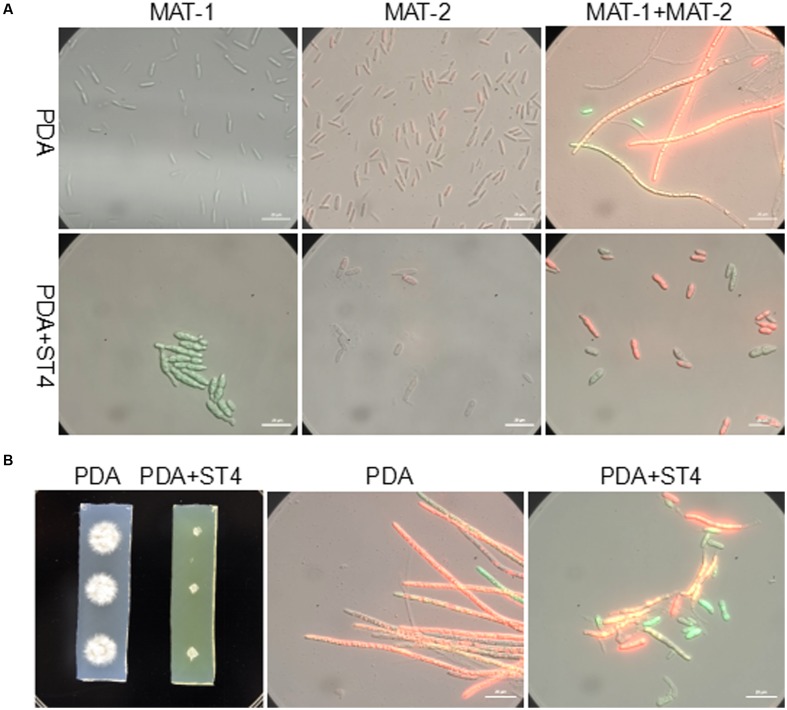
**Effects of ST4 metabolites on sexual mating and growth of *S. scitamineum*.** **(A)** ST4 extracellular metabolites affected sexual mating of compatible haploid cells. MAT-1 and MAT-2, respectively, marked with GFP and RFP were used for visualization (Yan et al., 2016a). Morphology of bipolar sporida and hyphae was observed under 100× magnification using ZEISS Observer. Z1. Bar, 20 μm. **(B)** ST4 metabolites affected dikaryon hyphal growth after mating. Bar, 20 μm.

The ability of strain ST4 on inhibition of fungal mating was further tested by using metabolite plate of ST4, as we were not able to detect visible inhibitory activity using its culture supernatants. ST4 inhibitory metabolite plates were prepared by growing strain ST4 on PDA plate, removing bacterial cells after 2 days, melting the remaining medium at around 70°C and mixing with fresh PDA medium at 1:1 ratio. The control agarose plates were prepared in the same way by using strain DH5α to replace strain ST4, which did not show any inhibitory activity against the fungal mating (Supplementary Figure S1). The results showed that the mixture MAT-1 and MAT-2 didn’t produce white hyphae on the ST4 metabolite plate (**Figure 1C_1_**), and the colony morphology was similar to the corresponding haploid cell lines grew on the ST4 metabolite plates (**Figure 1C_3_**). In contrast, the MAT-1 and MAT-2 mixture produced white hyphal cells on the control *E. coli* metabolite agarose plate (**Figure 1C_2_**). In another set of experiments, we showed that haploid cell lines MAT-1 or MAT-2 on the ST4 metabolite plate (**Figure 1C_3_**) grew up in a way similar to the same cell lines on fresh PDA plates (**Figure 1C_4_**). To distinguish whether ST4 metabolites exactly affect mating of bipolar sporida or hyphal growth of the resultant dikaryon, we used visualized MAT-1 and MAT-2 haploid cells, respectively, (Yan et al., 2016a) to test the inhibition activity. Under microscopy, we observed that after ST4 treatment, the size of both MAT-1 and MAT-2 sporida expanded by over three times and the exosporia became rough and thicker, and almost no dikaryon hyphae were found with only 1.05% of mating rate, while 94.77% of the control (**Figure 2A** and **Table 1**). To determine whether ST4 affects hyphal growth, we transferred hyphae from PDA after mating to new plates of PDA and PDA+ST4 metabolites, respectively. Results showed that hyphae became fragmented, fractured, and vanished after ST4 treatment (**Figure 2B**). Taken together, the above results indicate that strain ST4 produces a metabolite(s) capable of inhibiting not only the sexual mating of *S. scitamineum*, but also the growth of haploid cells or dikaryon hyphae.

**Table 1:** Mating rates of haploid cells on PDA and PDA+ST4 metabolite plates.

Medium nos.	MAT-1	MAT-2	MAT-1+MAT-2	Total	Mating rate
PDA	5	3	145	153	94.77%
PDA+ST4	169	209	4	382	1.05%


### Effect of Glucose on Fungal Growth and Inhibitory Activity of Strain ST4 on the Sexual Mating of *S. scitamineum*

**FIGURE 3:**
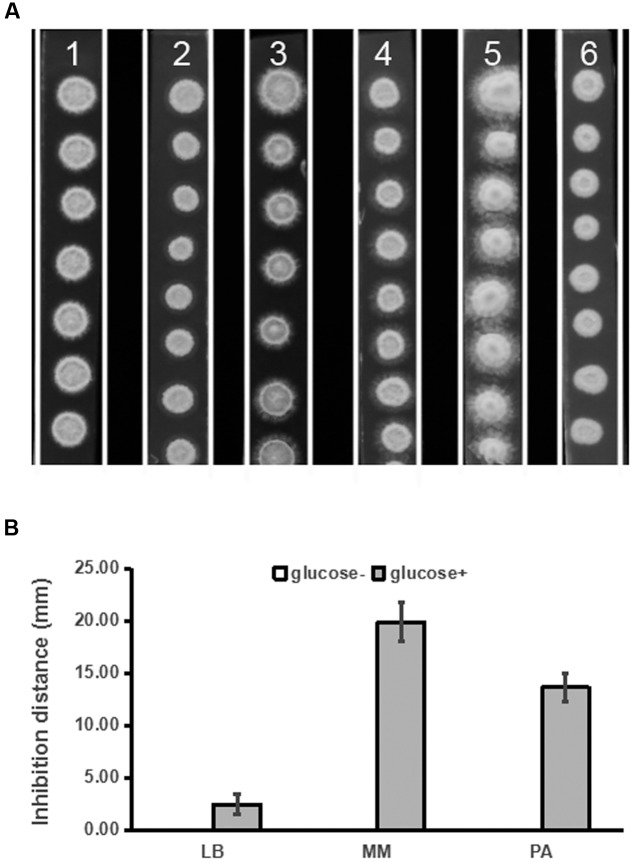
**Inhibitory effects of strain ST4 in different media on the sexual mating of *S. scitamineum*.** **(A)** *S. scitamineum* haploid cells MAT-1 and MAT-2. 1, LB medium+glucose; 2, LB; 3, MM medium+glucose; 4, MM; 5, PDA; 6, PA; **(B)** Inhibition distance of ST4 on the sexual mating of *S. scitamineum* haploid on different media.

In our preliminary study, we found that PDA medium was superior to LB, MM, and PA media for detection of inhibitory activity of strain ST4 against the fungal sexual mating. Given that PDA differs from PA by containing glucose, we tested whether addition of glucose to various media could improve production of mating inhibitory molecule(s) by strain ST4. The results showed that supplementation of glucose at a final concentration of 20 g per liter in LB, MM and PA media could improve hyphal growth of the fungal pathogen (**Figure 3A**), and could also substantially increase the production of mating inhibitory compounds (**Figure 3B**).

**FIGURE 4:**
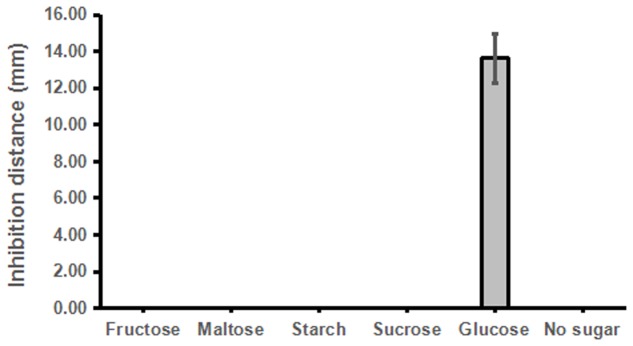
**Effects of different sugar sources on the sexual mating of *S. scitamineum***.

Similarly, we then tested whether other sugar or sugar derivatives could promote strain ST4 to produce inhibitory compounds, using glucose as a positive control. The results showed that only glucose could support strain ST4 to produce inhibitory compounds against the fungal sexual mating, whereas sucrose, maltose, fructose, and starch had no any effect (**Figure 4**).

**FIGURE 5:**
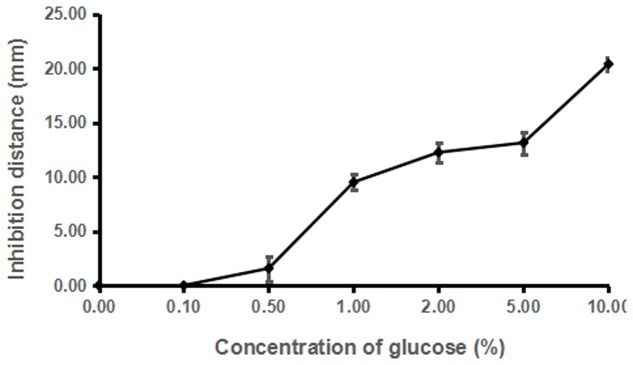
**Effects of different concentrations of glucose on the sexual mating of *S. scitamineum***.

Dosage analysis showed that the inhibitory activity of strain ST4 was enhanced along with the increased concentration of glucose from 1% till 8%, but no inhibitory activity could be detected when glucose concentration was at 0.5% (**Figure 5**). In a separate experiment, we found that addition of glucose alone in the same concentration range as in **Figure 5** without strain ST4 could not inhibit the fungal mating (data not shown), suggesting that glucose might be used as a substrate by strain ST4 in production of inhibitory compounds.

### Evaluation of the Biocontrol Potential of Strain ST4

**FIGURE 6:**
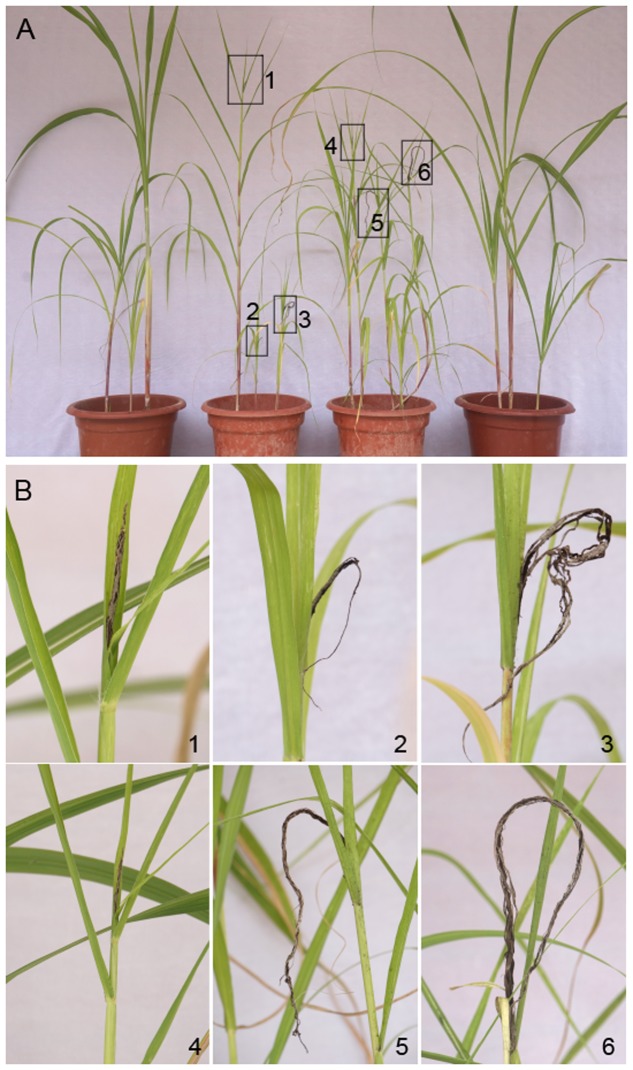
**Effect of strain ST4 on biocontrol of sugarcane smut disease.** **(A)** Symptoms of whole plants treated with ST4+*S. scitamineum*+2% glucose, ST4+*S. Scitamineum*, *S. scitamineum* (positive control), and PBS buffer (negative control) from left to right. **(B)** Smut spikes of sugarcane corresponding to the outlined in frame in **(A)**.

The biocontrol potential of strain ST4 was determined under greenhouse conditions. The results showed that the smut disease symptoms became visible at about 3 months after inoculation of mixed fungal cell lines MAT-1 and MAT-2 (**Figures 6A,B**). In contrast, addition of strain ST4 and glucose in the fungal inoculation mixture blocked smut disease symptom development (**Figures 6A,B**). Consistent with the results of plate bioassay (**Figure 4**), addition of strain ST4 alone without glucose in the inoculation mixture had no effect on biocontrol of the smut disease (**Figures 6A,B**). As expected, the sugarcane seedling showing smut disease symptoms grew poor than the control plants without inoculation and those treated with strain ST4 and glucose (**Figure 6A**).

Quantitative analysis of the disease rate (**Table 2**) showed that smut incidence was maximum in pots in which sugarcane sets were planted after inoculation of the fungal lines MAT-1 and MAT-2, following by the pots in which sugarcane sets was inoculated with the mixture containing MAT-1, MAT-2, and strain ST4, but the smut incidence was significantly reduced by about 65% when the MAT-1/MAT-2/ST4 mixture was inoculated in the presence of glucose (2%, wt/vol).

**Table 2:** The effect of strain ST4 on control of sugarcane smut disease.

Treatment	Sugarcane smut	
	
	Incidence (%)	Control efficiency^a^ (%)
PBS control	0.00	–
positive control	77.78	–
ST4+*S. scitamineum*	72.73	6.49
ST4+*S. scitamineum*+2% glucose	13.04	83.23


*^a^Control efficiency (%) = (Incidence_*positive control*_ - Incidence_*treatment*_) × 100/Incidence_*positive control*_.*

### Characterization of Strain ST4

**FIGURE 7:**
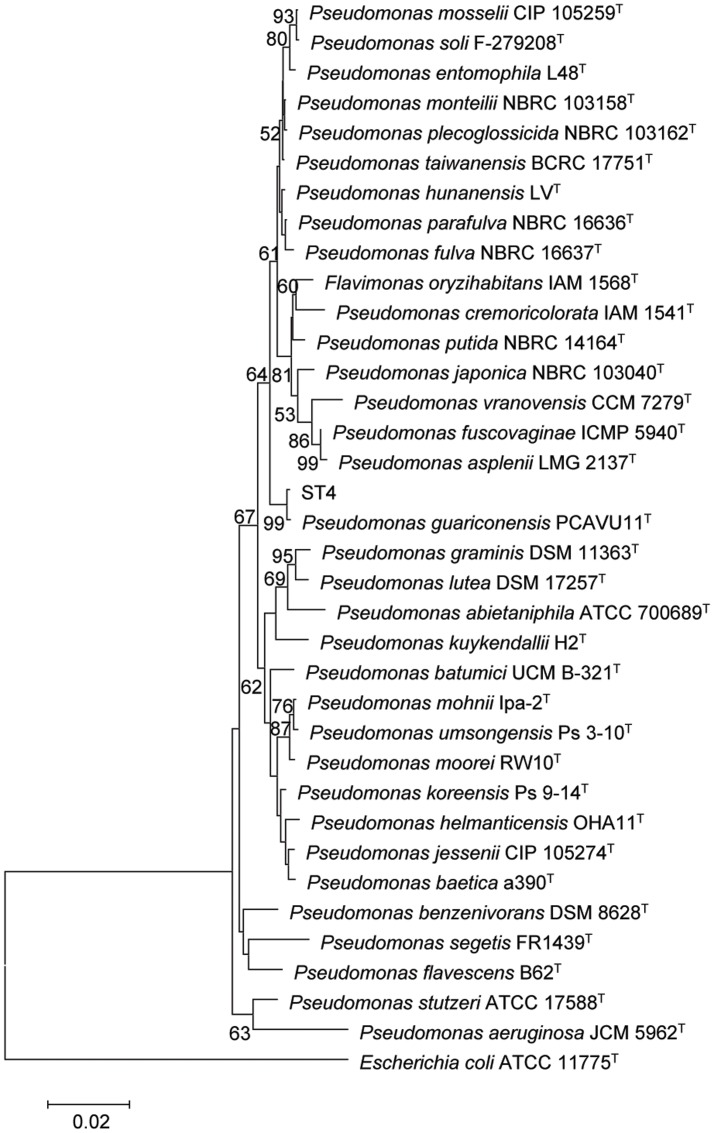
**Neighbor-joining tree showing the 16S rRNA gene phylogenetic relationships of strain ST4 and phylogenetically related reference strains.** Bootstrap values based on 1,000 resamplings are shown at branch nodes. Bar, 0.2% substitution rate per site.

**FIGURE 8:**
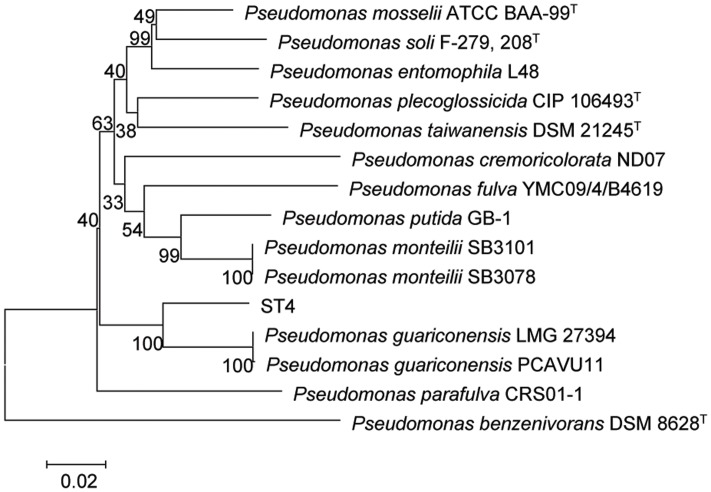
**Neighbor-joining tree showing the *gyrB*, *rpoB*, and *rpoD* gene phylogenetic relationships of strain ST4 and phylogenetically related reference strains.** Bootstrap values based on 1,000 resamplings are shown at branch nodes. Bar, 0.2% substitution rate per site.

The strain ST4 was isolated from vegetable rhizosphere in Shantou city, Guangdong province, China. Its colonies on LB plate are beige, translucent, convex, round, and smooth with entire margins. Electron micrograph showed that cells are straight rods, motile by means of polar flagella, with wrinkle cell surfaces (Supplementary Figure S2). Analysis of the 16S rRNA gene sequence revealed that strain ST4 belongs to the genus of *Pseudomonas*, showing high similarities to *P. guariconensis* (99.79%), *P. taiwanensis* (99.22%), *P. monteilii* (99.14%), *P. plecoglossicida* (99.07%), *P. mosselii* (99%), *P. entomophila* (99%), and *P. putida* (98.22%) (Supplementary Table S1). Phylogenetic analysis based on 16S rRNA gene sequence with neighbor-joining approach indicated that the isolate ST4 was clustered together with *P. guariconensis* PCAVU11^T^ (**Figure 7**). We further determined the partial gene sequences of three other housekeeping genes, i.e., *gyrB*, *rpoB*, and *rpoD*, of strain ST4, which share 94.25, 94.71, and 93.69% identity to their counterparts in *P. guariconensis*, respectively (Supplementary Table S1). The reconstructed phylogenetic tree based on the partial *gyrB*, *rpoB*, and *rpoD* gene sequences indicated that strain ST4 was clustered together with *P. guariconensis* PCAVU11^T^ and *P. guariconensis* LMG 27394 (**Figure 8**).

Further biochemical tests showed that strain ST4 is Gram negative and strictly aerobic. Optimum temperature for growth is 28°C, grew at 44°C, pH range for growth is 5–10, could grow with 0–5% NaCl in LB broth. The DNA G+C content was 63.56 mol%. A diffusible fluorescent pigment is produced on King’s B medium, which is the same as *P. guariconensis* PCAVU11^T^ (**Table 3**). Positive for arginine dihydrolase, urease, oxidase, and catalase; but negative for nitrate reduction, aesculin hydrolysis, indole and β-galactosidase production. In Biolog GN III plates, strain ST was positive in assimilation of D-glucose, D-fructose, glycerol, L-arginine, L-histidine, L-pyroglutamic acid, L-serine, L-aspartic acid, L-glutamic acid, L-lactic acid, citric acid, D-malic acid, α-keto-glutaric acid, L-malic acid, bromo-succinic acid, β-hydroxy-D, D-gluconic acid, L-butyric acid, propionic acid and amino-butryric acid. However, strain ST4 could not utilize sucrose, D-turanose, stachyose, gelatin, *N*-acetyld-D-glucosamine, *N*-acetyld-β-D-glucosamine, α-hydroxy-butyric acid, *N*-acetyl neuraminic acid, D-cellobiose, D-galactose, gentiobiose, lactose, maltose, D-mannitol, D-mannose, D-melibiose, L-rhamnose, D-trehalose, D-saccharic acid, D-glucose-6-phosphate, β-methyl-D-glucosamine, mucic acid, D-salicin, 3-methyl glucose, D-sorbitol, glycyl-L-prolin, α-keto-butyric acid, *N*-acetyl-D-galactosamine, D-raffinose, D-arabitol, *m*-inositol, D-aspartic acid, acetoacetic acid and formic acid. Reactions to dextrin, D-serine, tween 40, D-fucose, L-fucose, inosine, D-fructose-6-phosphate, L-alanine, pectin, D-galacturonic acid lactone, D-glucuronic acid, glucuronamide, methyl pyruvate, D-lactic acid methyl ester and acetic acid were variable (**Table 3**). The above morphological and biochemical properties of strain ST4 are reminiscent but not identical with those of *P. guariconensis* strain PCAVU11 (**Table 3**). In combination of the phylogenetic analysis and the results of biochemical test, we recommend assigning strain ST4 as *P. guariconensis*.

**Table 3:** Phenotypic features of strain ST4 and phylogenetically related strain.

Characteristic	*P. guariconensis* PCAVU11^a^	ST4
Flagellation	Two polar	Two polar
Fluorescent pigments King’s B agar	+	+
Growth at 44°C	+	+
Growth at 5% NaCl	+	+
(G+C) mol %	61.50	63.56
Assimilation of		
Indole production	-	-
β-Galactosidase production	-	-
Arginine dihydrolase	+	+
Urease	+	+
Nitrate reduction	-	-
Aesculin hydrolysis	-	-
Glucose	+	+
Gluconate	+	+
Caprate	+	+
Malate	+	+
Citrate	+	+
L-arabinose	-	-
Mannitol	-	-
*N*-acetylglucosamine	-	-
Phenylacetate	-	-
Adipate	-	-
Glycerol	+	+
Dextrin	-	V
D-maltose	-	–
D-trehalose	-	–
D-cellobiose	-	–
Gentiobiose	-	–
Sucrose	–	–
D-turanose	–	–
D-raffinose	–	–
D-lactose	–	–
D-melibiose	–	–
β-Methyl-D-glucoside	–	–
D-salicin	V	–
D-mannose	–	–
D-fructose	V	+
D-galactose	–	–
L-rhamnose	–	–
Inosine	+	V
D-sorbitol	–	–
L-aspartic acid	+	+
D-arabitol	–	–
Myo-Inositol	–	–
D-serine	–	V
L-alanine	+	V
L-glutamic acid	+	+
L-histidine	+	+
L-serine	+	+
Tween 40	+	V
Methylpyruvate	+	V
β-Hydroxybutyrate	+	+
α-Ketoglutarate	+	+
L-lactate	+	+
Propionate	+	+
L-pyroglutamate	+	+
γ-Aminobutyrate	+	+
*N*-acetyl-D-galactosamine	–	–
*N*-acetyl-D-glucosamine	–	–
L-fucose	–	V
Formate	–	–
Galactonate lactone	–	–
α-Hydroxybutyrate	–	–
*p*-hydroxyphenylacetate	–	–
α-Ketobutyrate	–	–
Glucuronamide	–	V
Glucose-6-phosphate	–	–


*^a^The data of strain PCAVU11 were from Toro et al. (2013). Symbol: -, negative; +, positive; V, variable.*

## Discussion

Rhizosphere is a region full of benificial or deleterious microorganisms, and is the first defense line from the attack by soilborne pathogens (Weller, 1988). Therefore, screening rhizosphere-competent bacteria is an excellent way to biocontrol microbial pathogens.

In this study, we isolated a bacterial strain ST4 from the rhizospheric soil sample in Guangdong, China. Strain ST4 showed a potent inhibitory effect against the sexual mating of *S. scitamineum*, while allowing growth of its haploid cells. We further showed that strain ST4 provided effective biocontrol against sugarcane smut disease under greenhouse conditions. To circumvent the environmental hazards and residual problems associated with application of chemical fungicides, plant and fungal extracts, which acted through inhibition of fungal mycelial growth and teliospore germination, were tested to control sugarcane smut disease with some effect (Lal et al., 2009). The results from this study showed for the first time, to the best of our knowledge, that effective biocontrol of sugarcane smut disease could also be achieved by using a bacterial isolate to not only inhibit the growth of haploid sporida and hyphae, but also interfere the fungal sexual mating.

As widely known, the recognized mechanisms of biocontrol include competition for niches or substrates, production of inhibitory compounds or cell wall degrading enzymes, and induction of systemic resistance (Hayat et al., 2012). Our results in plate assays showed that the potential mechanism of ST4 is to secret some inhibitory compounds to affect the sexual mating of MAT-1 and MAT-2 (**Figure 2A**, **Table 1**, and Supplementary Figure S3) and the growth of sporida and hyphae (**Figure 2**), and glucose was essential for strain ST4 to produce the fungal mating-inhibitory compounds and to control the sugarcane smut disease (**Figure 6** and **Table 2**). This is intriguing as among a few saccharides tested, including sucrose, maltose, and fructose, none of them could support strain ST4 to produce the fungal mating-inhibitory compounds. Given that the inhibitory activity of strain ST4 was increasing along with the increment of glucose concentration, we reasoned that glucose or its metabolites could be used for biosynthesis of the anti-mating compounds by strain ST4. Purification and characterization of the inhibitory compounds and elucidation of the corresponding biosynthetic pathway shall shade a light to dissolve this intriguing puzzle.

Strain ST4 was identified as *P. guariconensis* based on genotypic, phenotypic and chemotaxonomic analyses. *P. guariconensis* is a newly identified bacterial species and only one report, which was on taxonomic characterization (Toro et al., 2013), can be found from the NCBI literature database. The type strain is PCAVU11 that was isolated from the rhizospheric soil in Venezuela during the course of screening for phosphate-solubilizing bacteria (Toro et al., 2013). Although separated geographically by a long distance, strains ST4 and PCAVU11 appeared to share high levels of similarity genetically and biochemically. As most of the phosphorus in soils is present in insoluble form (Abd-Alla, 1994), the ability of *P. guariconensis* in solubilizing phosphate may be an additionally valuable and useful tract for strain ST4 as a biocontrol agent.

In general, this is the first report on biocontrol of sugarcane smut disease through antagonism against the sexual mating and hyphal growth of *S. scitamineum* sporida by a bacterium. Although the specific mechanism of antagonism is still to be clear, we reasoned that glucose or its metabolites could be utilized for biosynthesis of the inhibitory compounds by strain ST4. Our primary results represented that mutation in a gene encoding a glucose dehydrogenase in ST4 by either Tn5 transposon insertion or gene deletion resulted in the loss of inhibition activity against the sexual mating of MAT-1 and MAT-2 (Supplementary Figure S4), which supports this speculation. As was shown in **Figure 2**, the morphology of both bipolar sporida was changed after ST4 metabolites treatment, especially that of MAT-2 (**Figure 2A**), possibly reducing the compatibility of the haploid cells, implicating the possible reason of the actions of ST4 on the sexual mating of *S. scitamineum.* Furthermore, different components extracted from strain ST4 showed complicated actions on *S. scitamineum*, including suppression of the hyphal growth such as component S-2-6, and inhibition of the sexual mating, such as component S-2-7 (Supplementary Figure S3).

## Author Contributions

SL and LZ conceived and designed the experiments; JZ and YT helped to identify strain ST4; SL, NL, YmC, ZL, LL, ML, and YfC helped to perform the molecular work; FH and SC helped to analyze the active components produced by ST4; JZ and LZ revised the manuscript.

## Conflict of Interest Statement

The authors declare that the research was conducted in the absence of any commercial or financial relationships that could be construed as a potential conflict of interest.

## Footnotes

1 http://eztaxon-e.ezbiocloud.net/
