# Supplementary material for: Biocontrol of Sugarcane Smut Disease by Interference of Fungal Sexual Mating and Hyphal Growth Using a Bacterial Isolate
Source: Front Microbiol. 2017 May 9;8:778. doi: 10.3389/fmicb.2017.00778 (PMC5422470; doi:10.3389/fmicb.2017.00778)
Supplement: Supplementary file 1 [file Table_1.PDF]

Table S1. 16S rRNA, *gyrB*, *rpoB*, and *rpoD* gene sequence similarities between strain ST4 and type strains of phylogenetically related species.

Species	Gene sequence similarity (%) with strain ST4				
	16S rRNA	*gyrB*	*rpoB*	*rpoD*	concatenated partial *gyrB*, *rpoB* and *rpoD*
P. guariconensis	99.79	94.25	94.71	93.69	94.22
P. taiwanensis	99.22	89.00	93.56	84.57	89.04
P. monteilii	99.14	90.15	93.21	84.56	89.31
P. plecoglossicida	99.07	90.15	93.56	84.70	89.47
P. mosselii	99.00	91.95	93.90	86.61	90.82
P. entomophila	99.00	91.95	93.10	86.41	90.49
P. putida	98.22	89.98	91.48	84.35	88.60
P. fulva	98.22	86.86	91.94	82.52	87.11
