# Supplementary material for: Biocontrol of Sugarcane Smut Disease by Interference of Fungal Sexual Mating and Hyphal Growth Using a Bacterial Isolate
Source: Front Microbiol. 2017 May 9;8:778. doi: 10.3389/fmicb.2017.00778 (PMC5422470; doi:10.3389/fmicb.2017.00778)
Supplement: Supplementary file 2 [file Image_1.PDF]

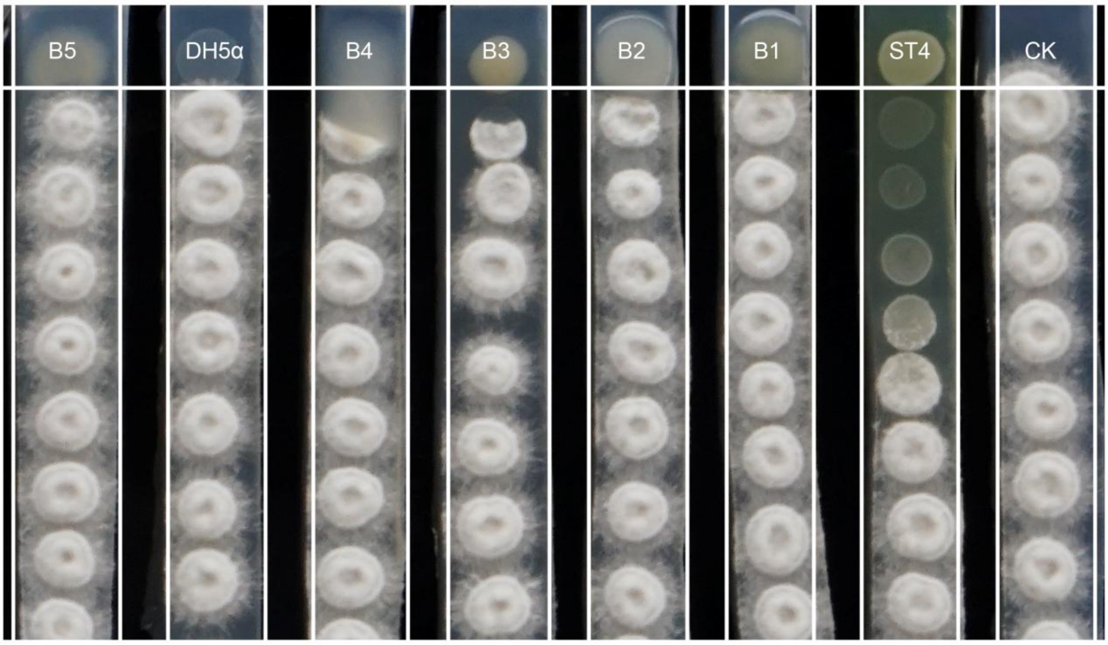

Fig. S1 Screening of biocontrol bacteria against mating of *S. scitamineum* by dual culture. For bioassay of inhibitory activity against the sex mating of *S. scitamineum*, the PDA plate was cut into separated slices (0.6 cm in width). An aliquot of 1 μl bacterial culture was added on one end of the agar slice, and then the mixture of *S. scitamineum* haploid cells MAT1 and MAT2 strain was spotted (0.5 μl of $OD_{600}\approx1.5$) on the slice at progressively further distances from the loaded sample. LB medium was added in the same way as bacterial culture as a negative control. The plates were incubated at 28 ^{o}C for 2 days, until the white hypha in the negative control grew to reach the edges of the slice.
