# Supplementary material for: Biocontrol of Sugarcane Smut Disease by Interference of Fungal Sexual Mating and Hyphal Growth Using a Bacterial Isolate
Source: Front Microbiol. 2017 May 9;8:778. doi: 10.3389/fmicb.2017.00778 (PMC5422470; doi:10.3389/fmicb.2017.00778)
Supplement: Supplementary file 3 [file Image_2.PDF]

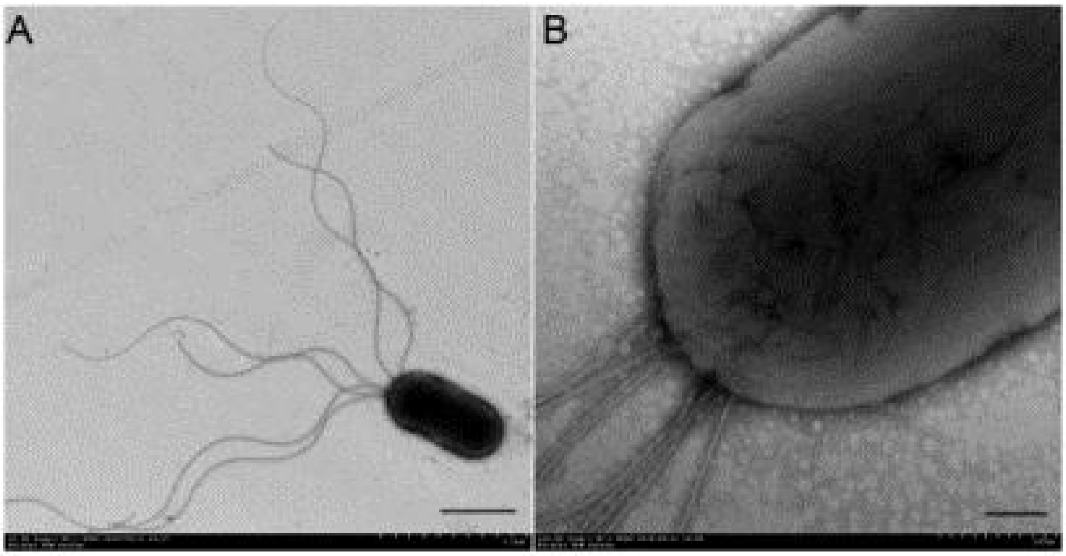

Fig. S2 Transmission electron micrograph of isolate ST4. Bacterial cells were suspended in sterile distilled water and stained with phosphotungstic acid [3 % (v/V), pH 7.0] for 2 min, air-dried and observed by using transmission electron microscope (Hitachi H7650). (A) Bar = 1.00 μm. (B) Bar = 100 nm.
