# Supplementary material for: Biocontrol of Sugarcane Smut Disease by Interference of Fungal Sexual Mating and Hyphal Growth Using a Bacterial Isolate
Source: Front Microbiol. 2017 May 9;8:778. doi: 10.3389/fmicb.2017.00778 (PMC5422470; doi:10.3389/fmicb.2017.00778)
Supplement: Supplementary file 4 [file Image_3.PDF]

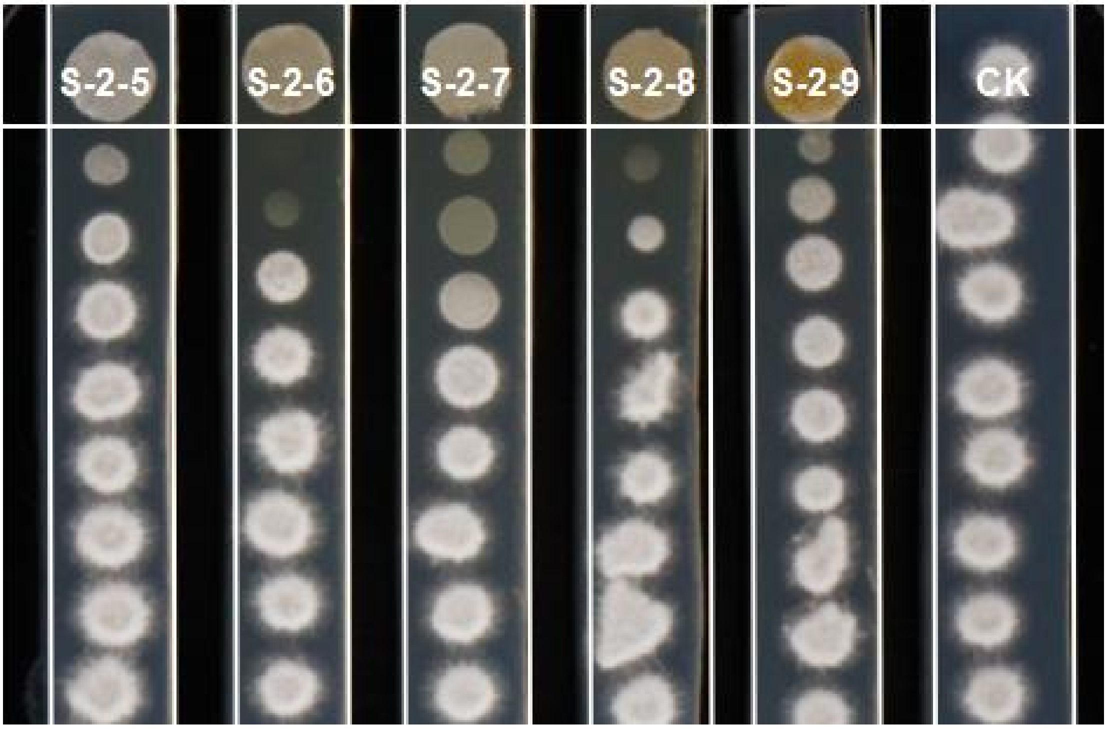

Fig. S3 Impacts of different components in ST4-2 fraction from strain ST4. ST4 was grown on PDA agar plates and chopped to sink in 3 times volumn of mixture ethyl acetate: methanol: glacial acetic acid (80:15:5) for 8-10 h. Supernatants were collected and ethyl acetate and methanol were removed using a rotary evaporator. The aqueous solution was extracted three times with 3 volumes of ethyl acetate, and organic phase from which was evaporated to dryness and concentrated under 45 ^{o}C, and dissolved in methanol, and separated using a Claricep FlashSilica (CS) standard silica gel column in different proportions of chloroform and methanol mixture solution. Component S-2 was then performed for HPLC separation. Results showed that metabolites from ST4 were rich in antibacterial active ingredients, some of which can inhibit the sexual mating of *S. scitamineum* sporida and inhibit the growth of mycelium such as S-2-8; and some of the components can inhibit the the sexual mating of *S. scitamineum* sporida with (S-2-7) or growth (S-2-6).
