# Supplementary material for: Biocontrol of Sugarcane Smut Disease by Interference of Fungal Sexual Mating and Hyphal Growth Using a Bacterial Isolate
Source: Front Microbiol. 2017 May 9;8:778. doi: 10.3389/fmicb.2017.00778 (PMC5422470; doi:10.3389/fmicb.2017.00778)
Supplement: Supplementary file 5 [file Image_4.PDF]

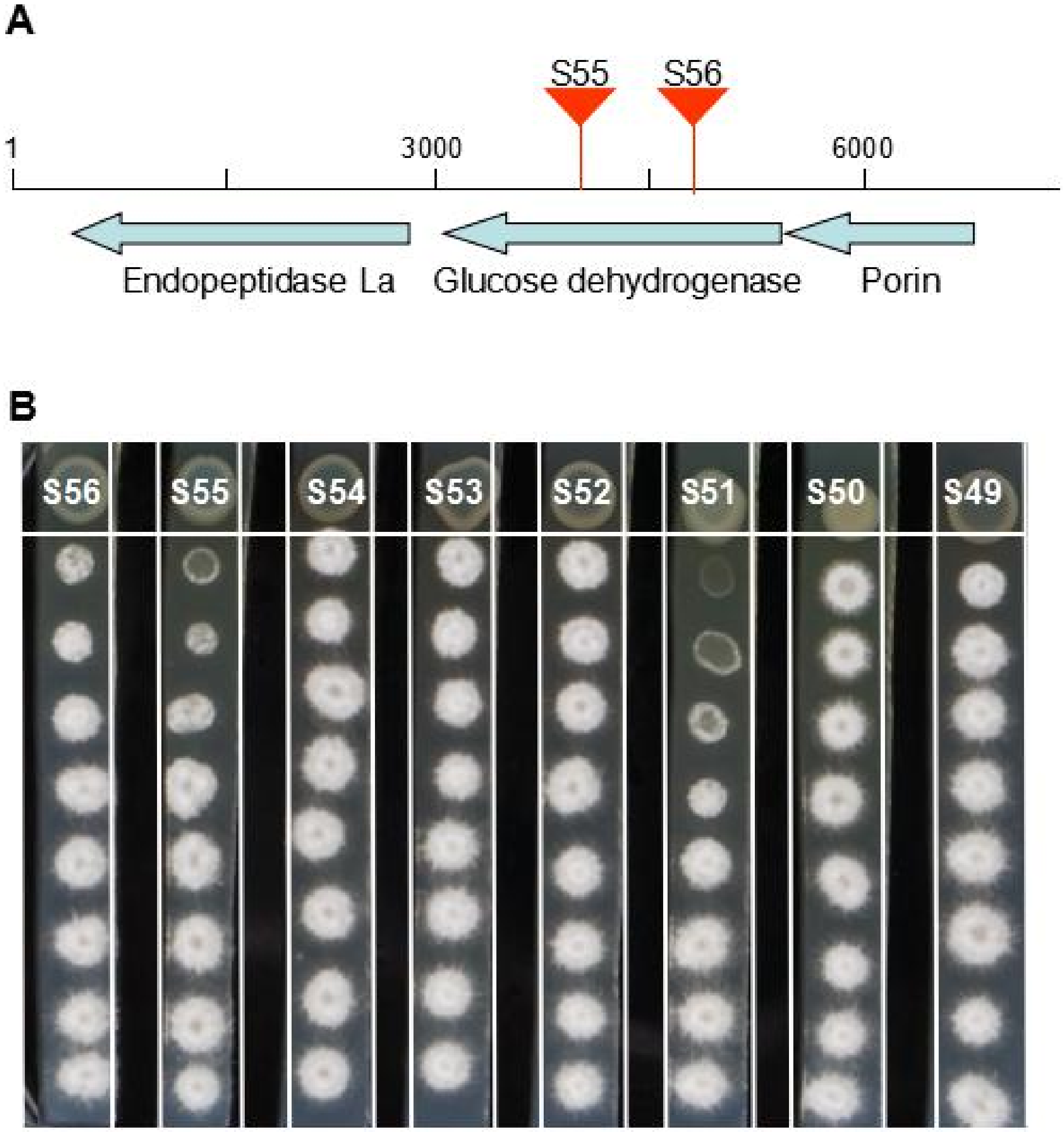

Fig. S4 Tn5 transposon insertions in the gene encoding a glucose dehydrogenase in ST4 lose the inhibition activity on sexual mating of *S. scitamineum*. (A) The genome organization of the gene encoding a glucose dehydrogenase in ST4. The relative transposon insertion sites and transcriptional directions were indicated. (B) Sexual mating inhibition activity of transposon mutants of ST4. S55 and S56 mutated in the glucose dehydrogenase encoding gene lose the inhibition activity.
